# Hydrophobia

**Published:** 1881-05

**Authors:** 


### HYDROPHOBIA.

THE gardener of A. A. A., Esq., heard his neighbor of Fifth Avenue, say, on Tuesday, May 2(., that his son had been bitten on the cheek by a dog, and that he would not have had it to occur for ten thousand dollars. This statement made such a strong impression on the gardener's mind as to the fearful nature of the bite of a dog, that, on reaching home, he complained of being unwell, and went to bed; in a short time he felt darting pains running up his arm towards the shoulder; subsequently, on offering him a drink of water, Mr. A. heard him make a noise like the bark of a dog, accompanied with a shuddering. The symptoms increased apace, and he died in horrible agonies on Friday, the fourth day.

Nine months before, this man was bitten on the complaining arm, but it soon healed, and nothing more was heard of it. This shows in a striking manner, what an influence the mind may have on the body. It is possible that the gardener might have had the attack, if he had not heard the conversation, but it is not probable, because he was in his usual good health.

We should learn from this not to nurse our symptoms, nor to allow the mind to dwell on any bodily infirmity, but if we have any ailment to make every possible effort to divert the current of thought, by mixing in cheerful society, engaging in good doing, or by embarking in some occupation which, while it secures bodily activity, compels the mind away pleasurably to things which feed and engross it. Earnest industry is a necessity of our nature; there is no persistent good health without it. No man was made to be a loafer. The idler, the do-nothing, is a drone among his fellows, the curse of his kind. All have much to do. The time is short, and the night cometh, when no man can work. Be busy, then, one and all.

**MUSH BISCUIT.**—Make about a quart of Indian-meal mush or stirabout; while hot, add a piece of butter, about the size of an egg, thin it with milk, adding a little salt; then add some flour, thin it with a tea-cup of yeast, then add as much more flour as will make it the consistence of dough; knead it well, set it to rise, and bake with a hot fire. The meal makes the bread light, and thus removes the objection to the unhealthfulness of hot bread.

### A SURFEIT

In man is called founder in a horse, and is over-eating, eating more than the stomach can possibly convert into healthful blood. Wise men, and careful men will sometimes inadvertently eat too much, known by a feeling of fulness, of unrest, of a discomfort which pervades the whole man. Under such circumstances, we want to do something for relief; some eat a pickle, others swallow a little vinegar, a large number drink brandy. We have swallowed too much, the system is oppressed, and nature rebels, instinct comes to the rescue, and takes away all appetite, to prevent our adding to the burden by a morsel or a drop. The very safest, surest, and least hurtful remedy, is to walk briskly in the open air, rain or shine, sun, hail, or hurricane, until there is a very slight moisture on the skin, then regulate the gait, so as to keep the perspiration at that point, until entire relief is afforded, indicated by a general abatement of the discomfort; but as a violence has been offered to the stomach, and it has been wearied with the extra burden imposed upon it, the next regular meal should be omitted altogether. Such a course will prevent many a sick hour, many a cramp, colic, many a fatal diarrhœa.

---

### SPRING DISEASES.

A very large amount of the discomfort, lassitude, depression and actual sickness which prevails in the spring can be modified or wholly avoided by the exercise of a little reflection and self denial

One of the main objects of eating in winter time is to keep us warm, and to that end, provident nature gives a vigorous appetite in cold weather, but if as warm weather approaches we eat with the appetite of winter, the system not having time to adapt the appetite to the temperature, mischief will follow as inevitably as if a locomotive is diminished in speed one half while the fires are kept burning as fiercely as when it was moving at the utmost allowed rapidity. A prompt diminution at each meal of one-third of the amount eaten in winter, beginning before April in the latitude of New York, and earlier further south, would diminish spring diseases to an incalculable extent.

### THE LONGEST LIVERS

Are they who dwell in Palaces and Poor Houses. As contradictory, as this appears, it is not the less true. The reason of it is, in the fact, that, knowing they are provided for, the mind is at rest, and is wholly disencumbered of that eating anxiety, that care for to-morrow, which press so heavily upon the mass of mankind. The very rich and the very poor are not the healthiest; on the contrary, they are seldom entirely well; this indisposition takes away what little appetite a loafing life allows them, hence, for a short time, they eat almost nothing: this gives the stomach time to recuperate, while nature works off the surplusage, and by this double operation, they are made as well as ever in a few days. Hence, the best "Life Insurance" is to secure for yourself, at the earliest possible day, *a moderate, uniform, and* CERTAIN *income.*

